# Dr. Hullihen’s Essay on Abscess of the Jaws

**Published:** 1847-03

**Authors:** 


AN ESSAY ON ABSCESS OF THE JAWS. BY DR. HULLIHEN, OF WHEELING.

This little essay, by one of the most scientific dental surgeons of the West, appears to be well written, and relates to an important disease. We have looked over it with interest, but it is too exclusively special to afford anything, which extracted, would be interesting to our readers generally.

